# Electromechanically Coupled III-N Quantum Dots

**DOI:** 10.3390/nano13020241

**Published:** 2023-01-05

**Authors:** Daniele Barettin, Alexei V. Sakharov, Andrey F. Tsatsulnikov, Andrey E. Nikolaev, Nikolay Cherkashin

**Affiliations:** 1 Department of Electronic Engineering, Università Niccoló Cusano, 00133 Rome, Italy; 2 Ioffe Physico-Technical Institute RAS, 26 Polytekhnicheskaya Str., St. Petersburg 194021, Russia; 3 CEMES-CNRS and Université de Toulouse, 29 rue Jeanne Marvig, BP 94347, CEDEX 4, F-31055 Toulouse, France

**Keywords:** quantum dots, electromechanical fields, modeling, epitaxial layer growth

## Abstract

We exploit the three-dimensional (3D) character of the strain field created around InGaN islands formed within the multilayer structures spaced by a less than 1-nm-thick GaN layer for the creation of spatially correlated electronically coupled quantum dots (QDs). The laterally inhomogeneous vertical out-diffusion of In atoms during growth interruption is the basic mechanism for the formation of InGaN islands within as-deposited 2D layers. An anisotropic 3D strain field created in the first layer is sufficient to justify the vertical correlation of the islands formed in the upper layers spaced by a sufficiently thin GaN layer. When the thickness of a GaN spacer exceeds 1 nm, QDs from different layers under the same growth conditions emit independently and in the same wavelength range. When extremely thin (less than 1 nm), a GaN spacer is formed solely by applying short GI, and a double wavelength emission in the blue and green spectral ranges evidences the electromechanical coupling. With $\vec{k}\cdot \vec{p}$ calculations including electromechanical fields, we model the optoelectronic properties of a structure with three InGaN lens-shaped QDs embedded in a GaN matrix, with three different configurations of In content. The profiles of the band structures are strongly dependent on the In content arrangement, and the quantum-confined Stark effect is significantly reduced in a structure with an increasing gradient of In content from the top to the bottom QD. This configuration exhibits carrier tunneling through the QDs, an increase of wave functions overlap, and evidence emerges of three distinct peaks in the spectral range.

## 1. Introduction

High efficiency and high brightness monolithic and hybrid III-N semiconductor-based light-emitting diodes (LEDs) are the first candidates to replace all conventional light sources in the coming years. The development of novel crystal growth, design, and technology will pave the way to superior white LEDs targeted by a significant enhancement of light extraction along the entire process chain from semiconductor epitaxial layer growth to the application. To achieve this goal, internal and external losses in LEDs should be dramatically reduced, which requires the control of III-N semiconductor properties on a near-atomistic scale [1,2,3,4].

An active medium of modern LEDs based on InGaN/GaN materials is composed of thin two-dimensional (2D) layers serving as quantum wells (QWs), which are elastically strained and might be plastically relaxed because In(Al)GaN and GaN lattice parameters are largely mismatched. Carriers are free to migrate in the plane of a QW with a homogeneous distribution of indium and, thus, can easily reach and recombine at the cores of dislocations presenting in a high density in GaN material formed by epitaxy over a foreign substrate [5]. The composition and thickness of InGaN layers being limited to a maximum value of 30% and several nanometers, respectively, the maximum emission wavelength that can be reached is limited to 530 nm. A further increase in the indium composition within a QW leads to the phase separation effect, structure quality degradation and increased quantum Stark effect caused by a strong electric field arising in polar structures. As a result, the emission efficiency rapidly drops in the wavelength range of 550–570 nm [6]. A conventional solution widely used in the white light sources combines emission of blue/violet LEDs with that produced by a mixture of suitable phosphors excited by the light emitted from the light-emitting diode (LED) [7]. However, such a method is rather expensive and not environmentally friendly. Basically, quantum dots (QDs) might provide more effective localization of carriers preventing their transport toward defect areas and thus ensure a higher efficiency of LEDs despite a high dislocation density. Indium-rich 1–3 nm-large inclusions distributed within 2D InGaN layers were considered to serve as QDs [8,9]. Although providing better carrier localisation, an inhomogeneous indium distribution within an InGaN layer often induces local formation of additional dislocations that deteriorates LED efficiency [10]. Unfortunately, control over the composition and morphological parameters of such indium-rich inclusions is severely hampered and even their real existence was questioned [11]. As a result, most of the efforts undertaken by different groups are aimed at suppression of the indium-rich inclusions’ formation during epitaxy of InGaN layers. Such an approach assumes that these layers have to be immediately overgrown by (Al)GaN following their formation.

In many epitaxially grown semiconductor heterosystems, such as InAs/GaAs materials, QDs can be created in the form of 3D islands already during epitaxial deposition following the Stranski–Krastanov mode. The islands, which appear as a result of 2D-3D transformation driven by a gain in elastic energy, have a pyramid-like shape after their capping and an anisotropic strain and composition distribution [12,13], providing 3D confinement of charge carriers [14].

The 3D InGaN/GaN islands have also been thought to form by using a similar mechanism [15,16,17,18]. However, an alternative explanation for their formation has been proposed as well based on the experiment with the application of a growth interruption (GI) just after the formation of 2D InGaN QWs [19]. Initially, an enhanced efficiency of LEDs containing such an active region was attributed to the appearance, during GI, of indium-rich inclusions within the InGaN layers without the formation of additional dislocations. However, more recently, advanced TEM characterization of such structures revealed that growth interruption instead leads to the 2D layer surface corrugation [20]. The further development of the GI approach allowed us to reach a transformation of massive 2D layer into laterally extended (>50 nm large) well-separated InGaN islands. By monitoring the morphology, thickness, and composition evolution of QWs formed in this manner in comparison to immediately overgrown InGaN layers, a self-consistent scenario for the top-down transformation of a 2D InGaN layer into 3D islands during growth interruption has been proposed [21]. It was shown that the transformation of the 2D layer into islands is caused by a laterally inhomogeneous conversion of superficial InGaN into GaN. The presence of hydrogen in the atmosphere strongly amplifies this effect. The interaction of hydrogen with superficial indium atoms, which coat the growth surface, provokes their release from the InGaN layer surface. Metallic indium atoms’ evaporation and migration change local in-depth lateral and vertical composition of the InGaN layer that results in the formation of 3D InGaN islands embedded within a 2D GaN layer.

In spite of having large lateral size, these islands serve as QDs as was evidenced theoretically [22,23]. Effective localization of excitons is achieved within their central parts thanks to a 3D morphology and anisotropic strain distribution within and outside such islands. The formation of such QDs was demonstrated to provide an enhanced LED efficiency with respect to reference quantum wells emitting at the same wavelengths [21]. In contrast to technological methods provoking the formation of indium-rich inclusions, the growth interruption approach allows a control over morphological and composition characteristics of 3D islands and, eventually, over emission wavelengths of optoelectronic devices.

The creation of multilayer structures with QDs separated by tunnel-transparent barriers is an extremely promising approach for its application in the long-wavelength-emitting devices. The formation of common electronic states in electromechanically coupled QDs is predicted to allow the extension or manipulation of the wavelength emission range [22,24]. Moreover, the more effective elastic stress relaxation in a 3D island, compared to a 2D layer [12,23], opens a way to incorporate a larger amount of indium within an array of islands compared to a 2D layer. Thus, a range of possible emission wavelengths can be further extended.

Such an approach can become an effective alternative to the conventional solutions widely used for the fabrication of the white light sources like a combination of emission of a blue/violet LEDs with that produced by a mixture of suitable phosphors excited by the light emitted from the LED, a mixture of blue, green, red, and, if necessary, amber light emitted from various III-nitride and III-phosphide LEDs, and, eventually, vertical monolithic integration of two or more active regions emitting light at different wavelengths in the same LED structure [25].

The disadvantages of the monolithic multicolour LED structures containing two active regions, separated by spacers of various designs, emitting in the blue and green spectral range has been presented in Ref. [26]. The green QWs were grown on top of the blue ones. External quantum efficiency (EQE) of the studied structures was found to be systematically lower than those of the reference ones containing individual active regions only. This fact has been interpreted in terms of degradation of the materials’ quality in the green QWs grown on top of the blue QWs. The principal factor controlling the emission spectra of multicolour LEDs identified in that study was the electrical properties of the spacer separating different active regions. These factors were found to be especially valuable for the spacers as wide as 15 nm or more. The wider the spacer, the stronger is the doping impact on the contributions of individual active region to the total emission spectrum of a multicolour LED.

The monolithic approach that we propose in this paper allows us to avoid the principle issue related to the strongly different growth conditions required for the formation of green QWs on top of blue QWs and thus to avoid structural degradation of the materials. By reducing the spacer thickness to a value providing electron tunneling, the electrical sensitivity of the spacer to doping can be suppressed and wide emission from the sandwiched multilayer structure can be obtained, even at In composition inherent to blue QWs. The latter is controlled by optimizing the duration of GI in each layer. We have demonstrated some promising experimental results proving such a concept and a model which allows to explain the phenomenon. Recent theoretical calculations also confirm that a closely-coupled QD-and-QW structure will allow us to shift emission toward the long wavelength range [27]. Moreover, such structures can serve not only as an active medium for light-emitting devices but are also believed to be an appropriate candidate for realization of spin-polarized light source [28].

Due to interesting fundamental scientific issue and importance for a wide variety of applications, in this paper we propose to explore with an exhaustive model the particular property of strained 3D InGaN islands, namely inhomogeneous 3D strain distribution induced in the surrounding GaN matrix, for the creation of spatially correlated electronically coupled QDs. The paper is organized as follows. Section 2 describes some preliminary experimental results necessary to establish the feasibility of the studied structure. The simulation models are summarized in Section 3. Discussion of the computational results is given in Section 4. Section 5 completes the paper.

## 2. Preliminary Experimental Results

In order to establish an experimental confirmation of the promising theoretical prediction we are presenting in the following section, we have realized some preliminary experimental investigations.

**Figure 1:**
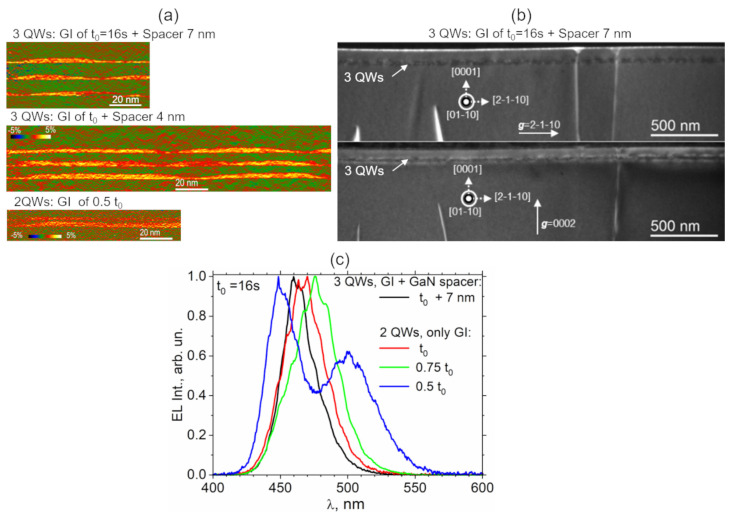
(**a**) Maps of vertical strain in InGaN vertically coupled islands obtained by dark-field electron holography in high-resolution mode. Upper image: three InGaN QWs, Gi t=16 s, 7-nm GaN spacer. Middle image: three InGaN QWs, Gi during t=16 s, 4-nm GaN spacer. Bottom image: two InGaN QWs, GI of 8 s, no intentional GaN spacer. (**b**) WBDF cross-sectional (01-10) images taken with g = 2-1-10 and g = 0002 of the same zone of the structure containing three QWs layers each subjected to a GI of 16 s and separated by a GaN spacer of 7 nm. (**c**) Normalized electroluminescence spectra from two InGaN QWs structures subjected to different time of GI compared to three InGaN QWs structure with a 7-nm-thick GaN spacer. Note two peaks in the spectrum of two InGaN QWs structure with GI of 8 s.

Our first objective is to verify whether it is possible to create vertically coupled InGaN islands. The second objective is to find a way to create a structure rather similar to that used in the model. Two sets of structures were grown by MOVPE by applying growth interruption approach developed in Ref. [20]. In the first set, the structures contain three InGaN layers, each subjected to GI during t=16 s following its formation and subsequently overgrown either by a 7-nm-thick or 4-nm-thick GaN spacer. In the second set, each structure contains two InGaN layers subjected to GI during different time of t=16 s, 12 s or 8 s. The second InGaN layer is deposited immediately after the completion of the GI applied to the first layer; that is, the InGaN layers are grown without intentional deposition of a GaN spacer in between. The two sets of samples have been analyzed from the point of view of their crystalline quality, morphology, composition, and electrical properties. Weak beam dark-field TEM imaging has been used to visualize dislocations at appropriate imaging conditions [29]. Except for the dislocations initially presenting in the GaN matrix, no additional dislocations in the active medium of either of the samples have been detected. Dark-field electron holography in a high-resolution mode is applied to extract the maps of out-of-plane strain in the studied structures with a 0.7–1 nm spatial resolution (Figure 1a) [30,31].

The formation of vertically correlated islands is detected in both sets of samples, i.e., even in the structure containing three layers of InGaN islands separated by the thickest GaN spacer of 7 nm. This finding confirms the presence of anisotropic 3D strain field induced by InGaN islands in the first layer being sufficient to induce a bottom-up vertical alignment of subsequent islands. A GI of 16 s is sufficient to produce quasiisolated 50-nm-large InGaN islands connected by a wetting layer (Figure 1a, two upper images). A two-times-shorter GI of 8 s in a two-QW structure still allowed the formation of islands that are connected by the thicker WL (Figure 1a, bottom image). In spite of the absence of intentionally deposited GaN spacer, it is worth noting the presence of a less than 1-nm-thick GaN spacer between the apexes of bottom islands and the WL of the upper islands in this structure. This fact evidences laterally inhomogeneous conversion of superficial InGaN into GaN during GI, which is in good agreement with experimental previous hypothesis [20].

Weak beam dark-field (WBDF) TEM imaging has been used to visualize dislocations at appropriate imaging conditions [29]. As an example, Figure 1b shows a couple of WBDF cross-sectional (01-10) images taken with g = 2-1-10 and g = 0002 of the same zone of the structure containing three QW layers, each subjected to a GI of 16 s and separated by a GaN spacer of 7 nm. Except for the dislocations initially presenting in the GaN matrix, no additional dislocations in the active medium of the sample have been detected. Such a result confirms a high crystalline quality of the active medium of all the studied structures.

Figure 1c shows the electroluminescence spectra obtained in the set of two QWs structures subjected to different time of GI in comparison with that obtained in three QWs structure with a 7-nm-thick GaN spacer. Except for the two QWs structure subjected to the shortest GI of 8 s, all spectra present one peak in the blue range. The peak position shifts in the shorter wavelength range with a duration of GI. The sample with a 7-nm spacer was effectively subjected to the longest GI if one considers some additional time before each QW was completely covered by the GaN layer. Accordingly, it presents the emission in the shortest wavelength range. The presence of a single emission peak in these structures indicates that each QW of the multilayer structure emits in the same wavelength range. Thus, in spite of being mechanically coupled in the vertical direction, the QDs are not connected electronically when the distance between InGaN multilayers is great enough. The change in QW indium composition explains the difference in the position of a single peak in the EL spectra of the samples that underwent different GI times. Indium atoms diffuse toward the free surface during GI, leaving behind a GaN structure that lowers the average indium content within a QW. The average In composition decreases with increasing GI. Thus, we observe a “blue” shift of the single peak position in the EL spectra of the samples with a duration of GI. When each of the shortest GIs of 8 s is subjected to the structure with double QWs, the GaN spacer formed above the first layer is so thin (less than 1 nm) that the QDs formed on both layers become electromechanically coupled. This causes the spectrum to split into two peaks, one in the blue range and the other in the green range. This is the first promising result demonstrating the feasibility to obtain a double-wavelength emission from electromechanically coupled multilayer III-N quantum dots. It should be noted that the external quantum efficiency (EQE) measured in the structure providing double wavelength emission has been found to be much smaller than that in the structures providing a single-peak emission. Thus, such structure is far of being optimized. Among still open questions, that of special importance is the role of absorption of light emitted from a short-wavelength QW by a long-wavelength QW and the impact of this effect on the total emission spectrum and efficiency of multicolour LEDs. Other important problems which we are dealing with and which will be presented in future articles already in preparation are how to control the electrical quality and the thickness of the GaN spacer which is formed during GI and a sophisticated control over the 3D morphology, composition distribution, density, and lateral size of InGaN islands formed under GI.

## 3. Models

Band structure calculations for our structures have been performed with an 8-band $\vec{k}\cdot \vec{p}$ model [32,33], with electron, heavy-hole, light-hole, and spin-orbit split-off bands described around the Γ point of the Brillouin zone and all other bands treated as remote bands. We used a 8×8 effective-mass Hamiltonian based on Foreman’s application of Burt’s exact envelope function theory to planar heterostructures [34,35], with parameters from Ref. [36]. The wave function of a state *n* with energy $E_{n}$ is a linear combination of the eight Bloch parts weighted by the respective envelope functions,

(1) $$\begin{array}{c} \psi_{n}=\sum_{i=1}^{8}\phi_{i}u_{i}, \end{array}$$

where $\phi_{i}$ are the envelope function and $u_{i}$ are the Bloch states [37].

The electromechanical field in the structures has been calculated by a fully coupled continuum model, as described in Refs. [38,39], with piezoelectric and strain fields which have been included in $\vec{k}\cdot \vec{p}$ model via deformation potentials [40].

As for the optical properties, we first calculated dipole matrix elements ${\vec{\mu }}_{nm}$ from the momentum matrix element within the 8-band envelope function approximation

(2) $$\begin{array}{cc} {\vec{p}}_{nm}\; & \equiv \langle \psi_{n}|\vec{p}\;|\psi_{m}\rangle \\ \; & =\sum_{i,j=1}^{8}\left(\langle \phi_{i}^{\left(n\right)}|\vec{p}\;|\phi_{j}^{\left(m\right)}\rangle \delta_{ij}+\langle \phi_{i}^{\left(n\right)}|\phi_{j}^{\left(m\right)}\rangle \langle u_{i}|\vec{p}\;|u_{j}\rangle \right)\\ & \equiv {{\vec{p}}_{nm}}^{\;(\phi )}+{{\vec{p}}_{nm}}^{\;(u)}, \end{array}$$

where ${\vec{p}}^{\;(\phi )}$ and ${\vec{p}}^{\;(u)}$ are the envelope and the Bloch parts of the momentum matrix element, respectively, where the envelope functions are assumed to be slowly varying at the scale of the primitive cell and are usually neglected [41,42], because they are much smaller than the Bloch parts.

Therefore, oscillator strengths are given by [43]

(3) $$\begin{array}{c} {\vec{P}}_{nm}=\frac{2\pi e^{2}\hbar^{2}}{\epsilon_{0}m_{0}^{2}V}{\left|\hat{e}\cdot {\vec{p}}_{nm}\right|}^{2}, \end{array}$$

where $\hat{e}$ is the direction unit vector of the electric field of the linearly or elliptically polarized incident light, *V* is the volume, *e* is the electron charge, $m_{0}$ is the free electron mass, and $\epsilon_{0}$ is the vacuum permittivity. All models are implemented and solved by using the TiberCAD simulator [44,45,46].

## 4. Optoelectronical Properties

**Figure 2:**
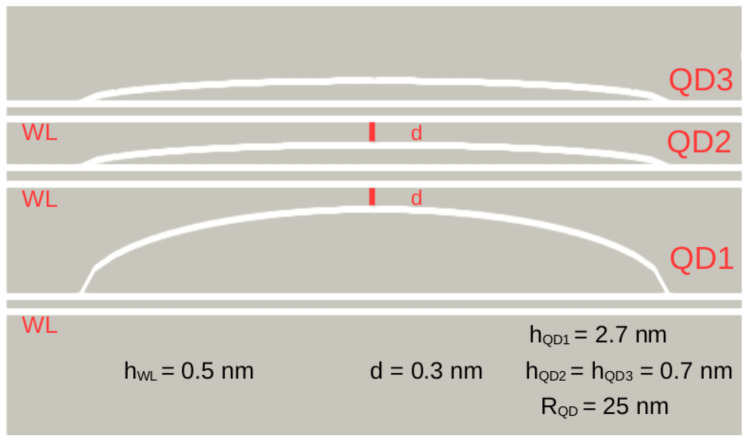
Model of the simulated structure with all geometric parameters.

It was shown in our previous paper that even obtaining mechanical autoorganization of InGaN islands in a multilayers structure, a GaN spacer thicker than 1 nm will prevent their electrical coupling [22]. Consequently, in order to assess the impact of electromechanical coupling on optical properties of LEDs with InGaN/GaN QDs in the active region, we have studied a model consisting of InGaN islands separated by extremely thin (d=0.3 nm) GaN spacers. More specifically, the structure contains three InGaN lens-shape island QDs embedded within GaN matrix one, just above the other and separated by two thin GaN spacers as shown in Figure 2. Each QD has a radius $R_{QD}=25$ nm. The first bottom island (QD1 in Figure 2) has a height $h_{QD1}=2.7$ nm. The second and the third ones have the same height $h_{QD2}=h_{QD3}=0.7$ nm. All the islands were constructed over a wetting layer (WL) with a thickness $h_{WL}=0.5$ nm. All geometric parameters are reported in Figure 2.

The model assumes constant In content inside each island, but each is different. Three levels of In content have been considered: low (L) of 15%, medium (M) of 20% and high (H) of 25%, and three different configurations of In bottom-up distribution within the QD have been simulated: HML, MMM, and LMH where the first, second, and the third letters stand for In content within the bottom (QD1), medium (QD2), and upper (QD3) islands, respectively. Consequently, three different situations have been studied regarding situations in which In content decreases, stays constant, or increases from the bottom to the top islands.

**Figure 3:**
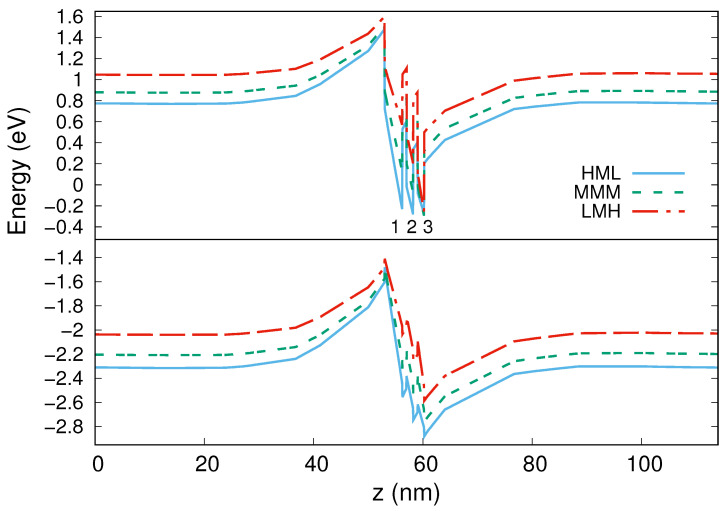
Conduction (**top**) and valence (**bottom**) band structures along vertical *z*-axis. Wells 1, 2, and 3 correspond to QD1, QD2, and QD3.

Figure 3 shows a plot of the band structures (the conduction band on top and valence band on bottom) along the vertical *z*-axis in the center of the QDs for the three configurations. We can observe that while the valence band profiles are quite similar, the profiles of conduction band are strongly dependent on the In content arrangement from bottom to top islands. It is well known that wurtzite structures possess huge built-in electric fields responsible for a quantum-confined Stark effect (QCSE) which produces conduction and valence band tilt and reduces the overlap of electron and hole wave functions, decreasing eventually the efficiency of radiative recombination [22,47,48].

For our structure in the LMH configuration, we note a relevant QCSE, while it decreases in the MMM and moreover in the HML configuration. Actually, the three QWs present practically the same confinement energy in the HML configuration. This could indicate an increase of confinement probability of the electrons in the lower part of the structure (QD1) and consequently an increase of overlap between electrons and holes wave functions.

**Figure 4:**
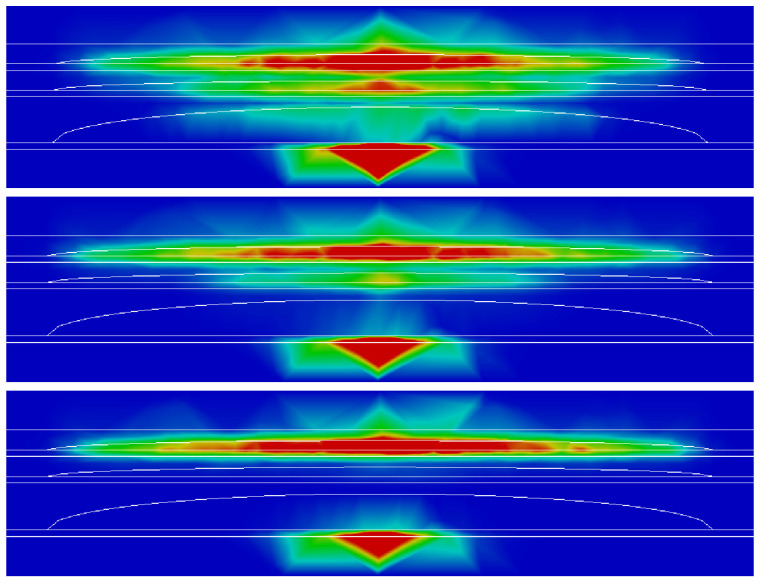
Linear combination of the probability densities of the first three holes states and of the first 100 electronic states in the xz plane calculated for the three different configurations (HML, MMM, LMH from top to bottom).

This finding is confirmed in Figure 4 which shows a linear combination of the probability densities of the first three holes states (below in the structure) and of the first 100 electronic states localized in the QDs in the xz plane calculated for the three different configurations (HML, MMM, LMH from top to bottom). In the HML configuration, the electrons are located throughout the structure, with a strong tunneling between the three islands. Tunneling becomes weaker in the MMM configuration, and completely disappears in the LMH structure. In this LMH configuration, electrons are confined in the upper island similar to what is usually found in a single InGaN QD.

**Figure 5:**
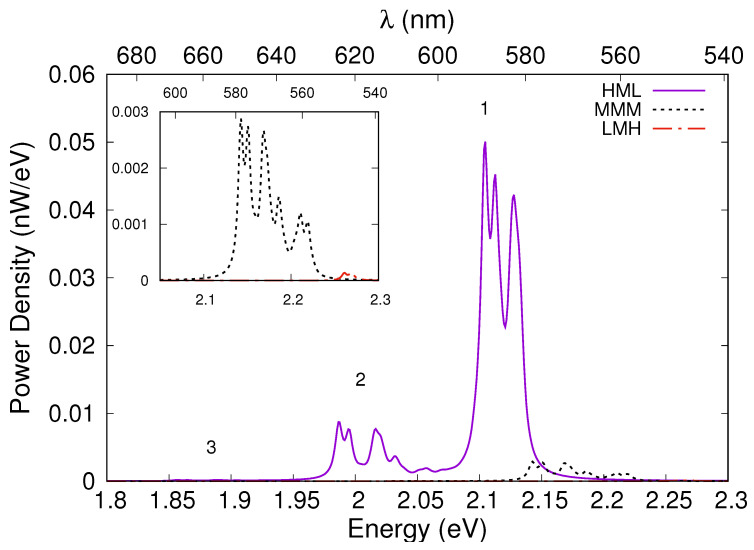
Spontaneous emission spectra for the HML, MMM, and LMH configurations. Inset: magnification of spontaneous emission spectra for the MMM and LMH configurations.

Spontaneous emission spectra calculated for these three configurations reveal the appearance of several peaks in the optical spectrum of the HML and MMM structures (Figure 5) that previously was possible to obtain only in monolithic multicolour LED structures containing two well-separated blue and green active regions [26]. The optical spectrum of the HML structure clearly presents three distinct peaks: one (starting from about 2.09 eV) corresponds to the transitions between the holes and the electrons confined in QD1; the second (starting from about 1.99 eV) corresponds to the transitions between the holes and the electrons confined in QD 2; a third, almost negligible, corresponds to the transitions between the holes and the electrons confined in upper QD3. The different intensity of the three peaks is clearly proportional to the overlap between the wave functions. The optical spectrum of the MMM structure is shifted to the higher energy range by about 0.1 eV and presents only the peaks 2 and 3, the latter again negligible. The LMH structure presents only one peak corresponding to the transitions between the holes and the electrons confined in island 3, which is again negligible compared to the others. To be more precise, in the inset of Figure 5, where we have a magnification of spontaneous emission spectra for the MMM and LMH configurations, we can clearly see that the peak corresponding to the MMM configuration has an intensity of more than an order of magnitude less than HML configuration, and that of LMH configuration at least two orders of magnitude less.

## 5. Conclusions

In this work, we have studied the possibility of exploiting multilayer structure of strained 3D InGaN islands embedded in a GaN matrix, for the creation of spatially correlated electronically coupled QDs.

As a first step, we have shown preliminary experimental results demonstrating the feasibility to achieve the vertical correlation of InGaN islands, formed by a growth interruption approach, in a multilayer structure. An anisotropic 3D strain field induced by InGaN islands in the first layer can induce a bottom-up vertical alignment of subsequent islands, provided the thickness of a GaN spacer between the layers is small enough. Electroluminescence spectra revealed peaks both in the blue and in the green spectral ranges, proving the possibility of obtaining double wavelength emission from electromechanically coupled multilayer III-N QDs.

Secondly, we have presented a model for the study of optoelectronic properties of a structure containing three InGaN lens-shape islands QDs embedded in a GaN matrix, with three different configurations of In bottom-up distribution. The profiles of the band-structures were found to be strongly dependent on the In content arrangement from bottom to top islands, and it was observed that QCSE is noticeably reduced in a structure with an increasing gradient of In content from the top to the bottom, resulting in a considerable tunneling of the carriers through the three QDs. The consequent increase of the overlap of the wave functions in this configuration results in the appearance of three distinct peaks in three different regions of the spectral range.

## Data Availability

The data presented in the current work are available on request from corresponding authors.

## Abbreviations

The following abbreviations are used in this manuscript:

LED	Light-emitting diodes
QWs	Quantum wells
QDs	Quantum dots
GI	Growth interruption
WBDF	Weak beam dark-field
EQE	External quantum efficiency
QCSE	Quantum-confined Stark effect
